# Supplementary material for: The Antioxidant and Prebiotic Activities of Mixtures Honey/Biomimetic NaDES and Polyphenols Show Differences between Honeysuckle and Raspberry Extracts
Source: Antioxidants (Basel). 2023 Aug 28;12(9):1678. doi: 10.3390/antiox12091678 (PMC10525646; doi:10.3390/antiox12091678)
Supplement: Supplementary file 1 [file antioxidants-12-01678-s001.zip › antioxidants-2534514-supplementary.pdf]

Supplementary Material

The Antioxidant and Prebiotic Activities of Mixtures Honey/Biomimetic NaDES and Polyphenols Show Differences between Honeysuckle and Raspberry Extracts

by Luminița Dimitriu, Diana Constantinescu-Aruxandei , Daniel Preda , Ionuț Moraru, Narcisa Elena Băbeanu
and Florin Oancea

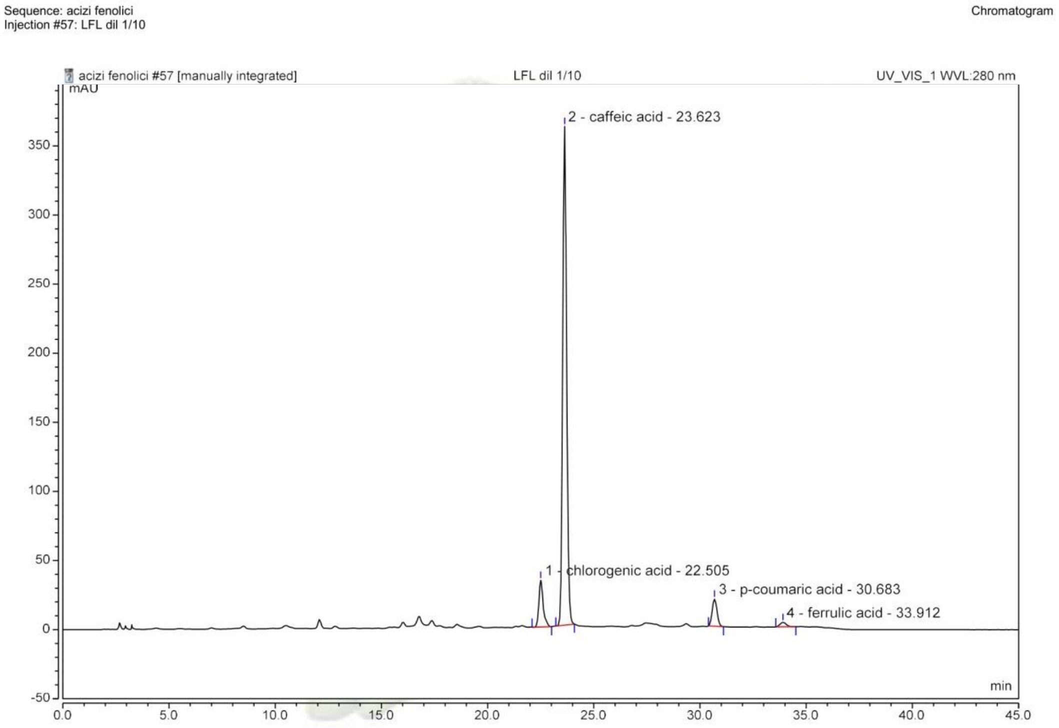

Figure S1. HPLC chromatogram of phenolic acids from honeysuckle flower extract

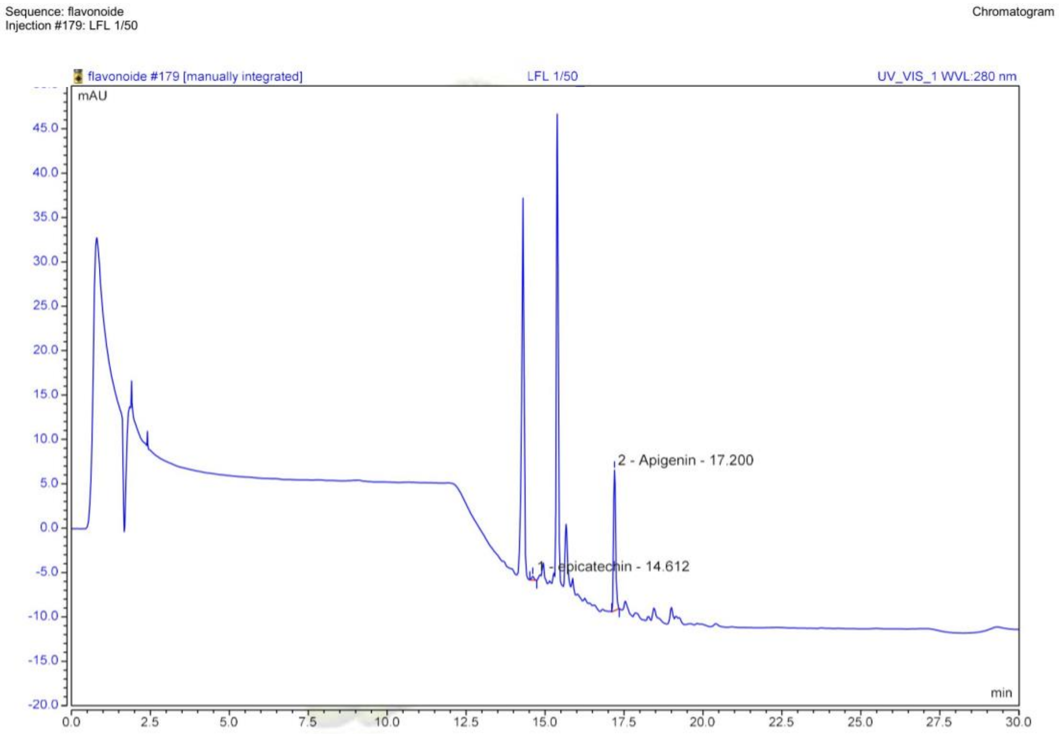

Figure S2. HPLC chromatogram of flavonoids from honeysuckle flower extract

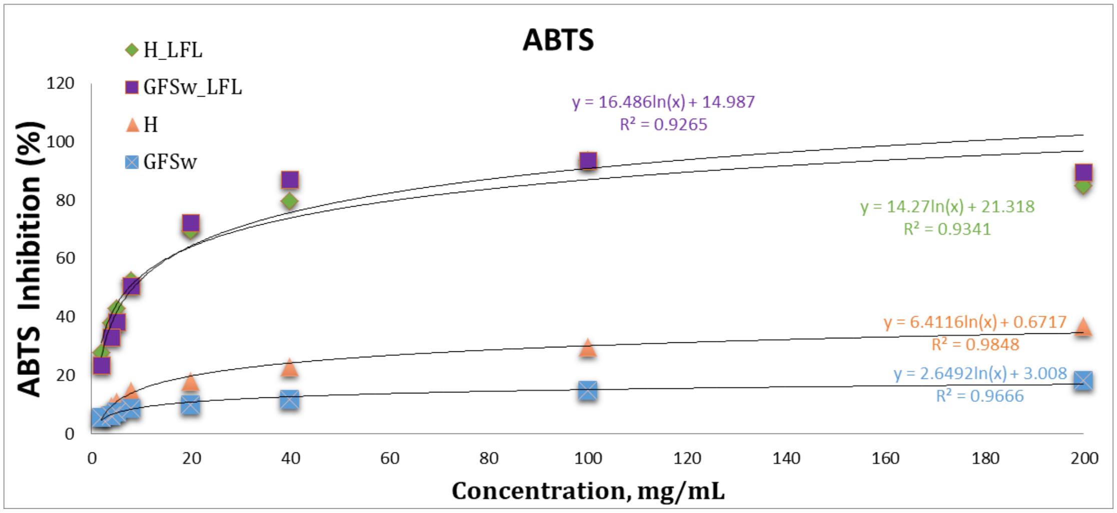

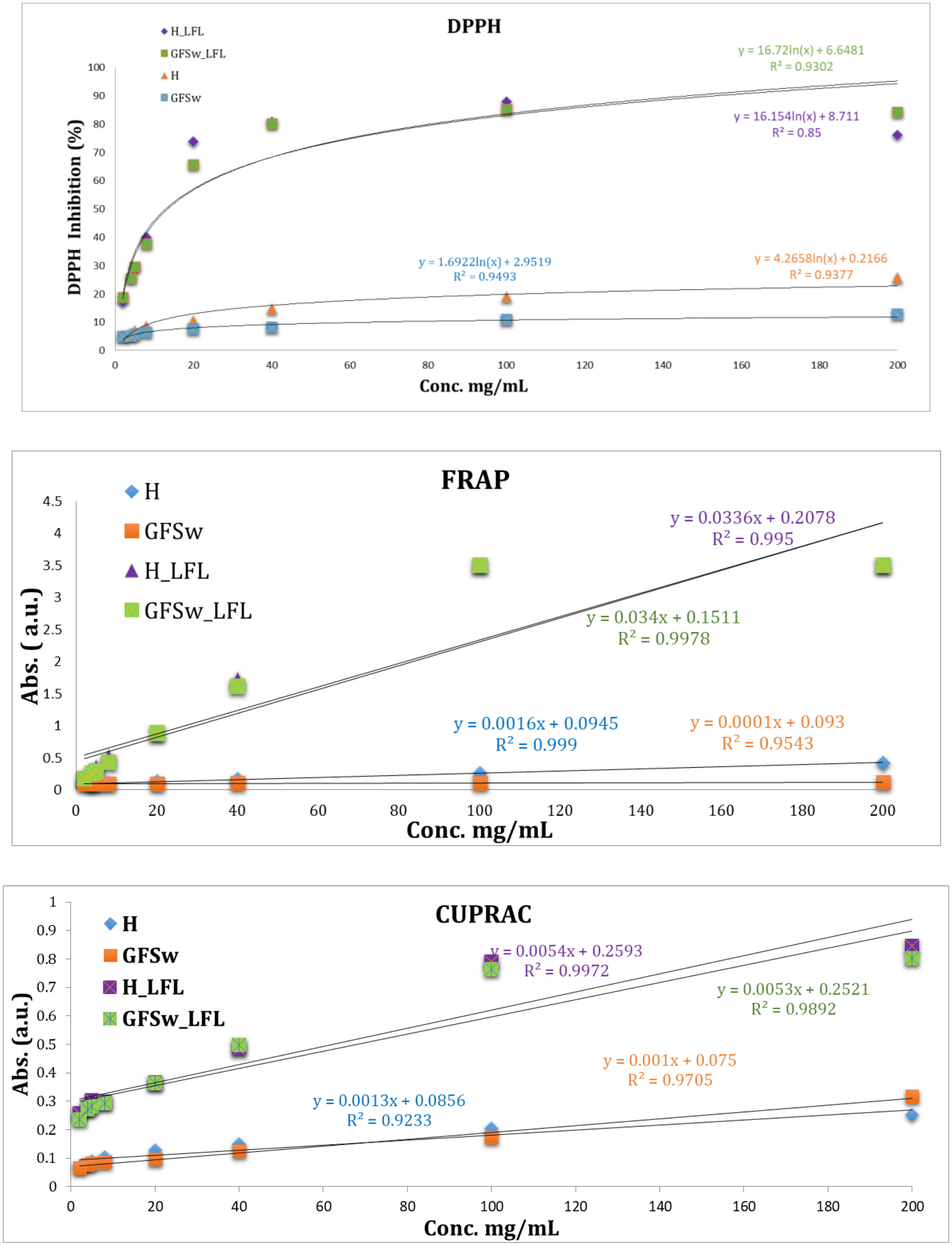

Figure S3. Dose-response curves for the AOA using DPPH, ABTS, FRAP, CUPRAC methods of the samples of honey (H), biomimetic NaDES with honey (GFSw), honey enriched with honeysuckle extract (H_LFL), GFSw enriched with honeysuckle extract (GFSw_LFL) at the concentration 2-200 mg/mL – it can be observed that the AOA of mixture of

honey and GFSw with honeysuckle extract decrease at concentrations greater than 100 mg/mL for DPPH, ABTS methods, and a little bit in the case of CUPRAC, in the case of FRAP methods the AOA cannot be measured at concentrations higher than 100 mg/mL (see main text)

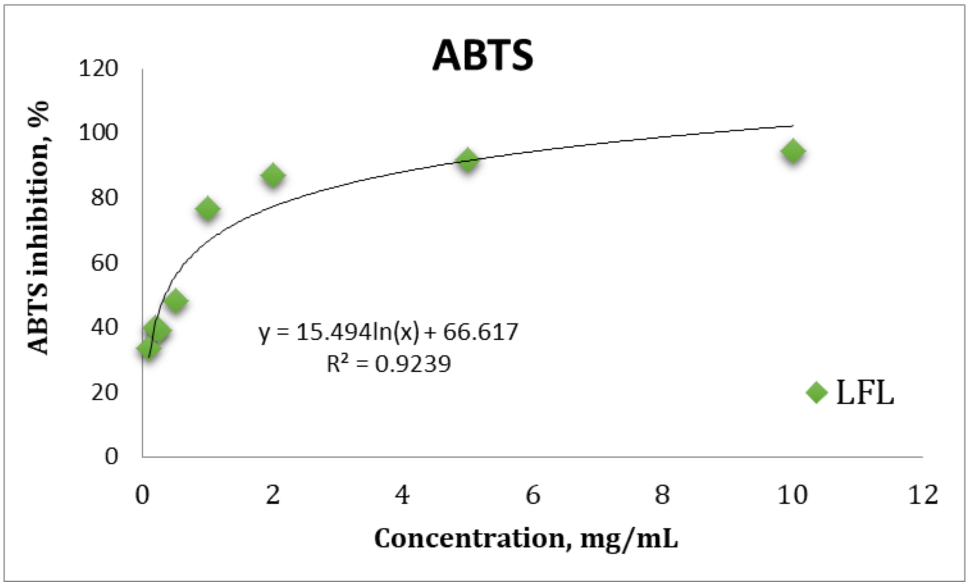

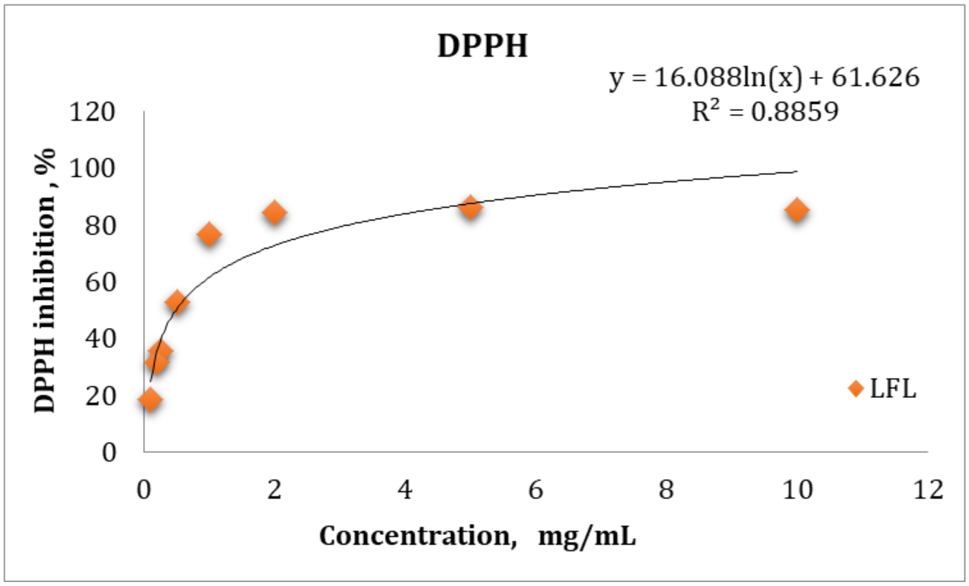

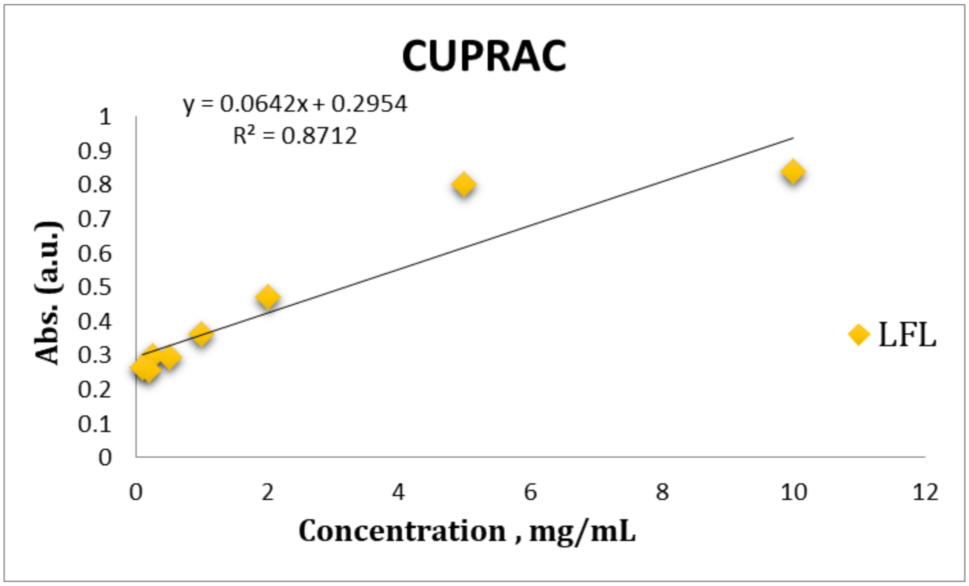

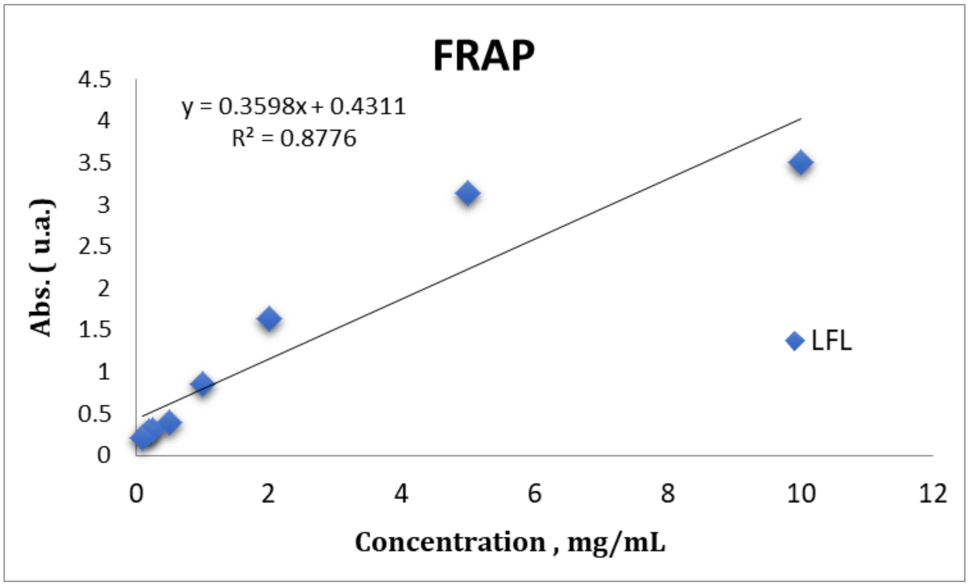

Figure S4. Dose-response curves for the AOA using DPPH, ABTS, FRAP, CUPRAC methods of the samples of honeysuckle extract (LFL) at the concentration of 0.1 - 10 mg/mL, in the case of FRAP and CUPRAC the curve was non-linear, and in the case of DPPH and ABTS the value decreases at the concentration 200 mg/mL

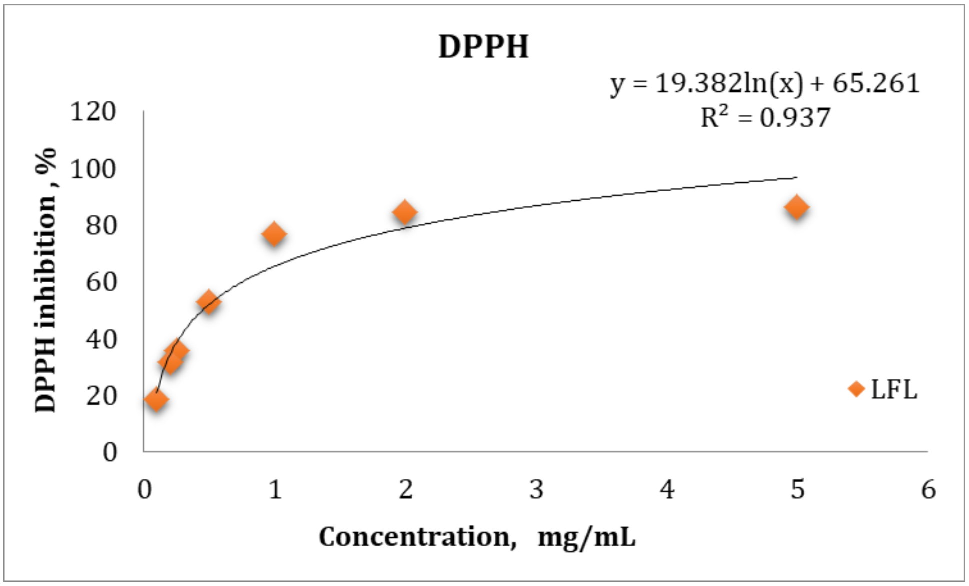
DPPH
y = 19.382ln(x) + 65.261
R² = 0.937
DPPH inhibition , %
120
100
80
60
40
20
0
0
1
2
3
4
5
6
LFL
Concentration, mg/mL

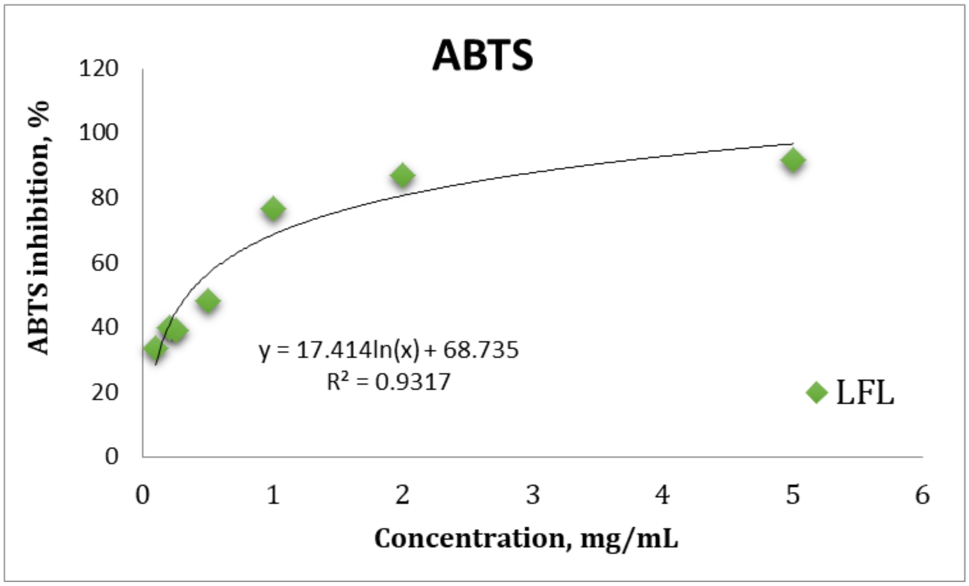
ABTS
ABTS inhibition, %
120
100
80
60
40
20
0
y = 17.414ln(x) + 68.735
R² = 0.9317
LFL
0
1
2
3
4
5
6
Concentration, mg/mL

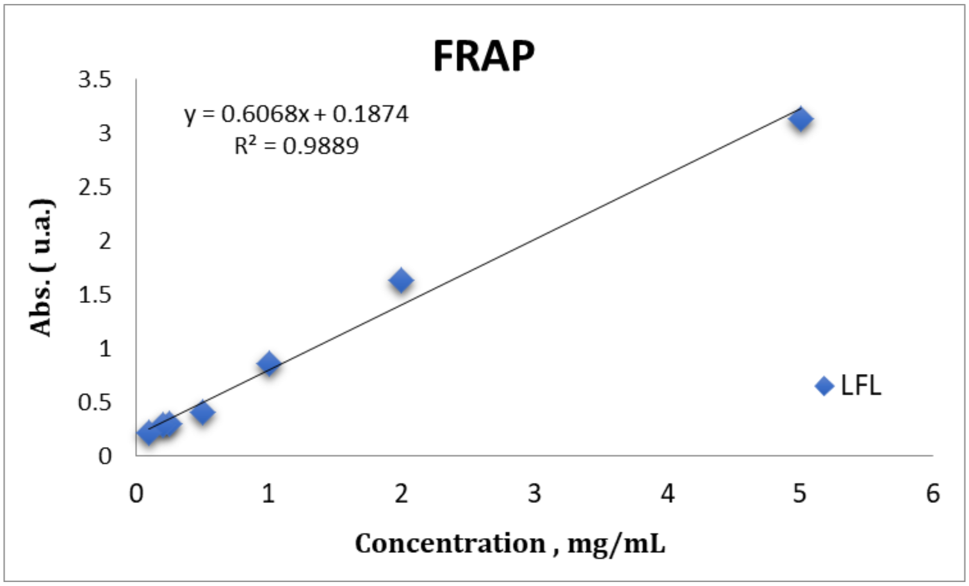

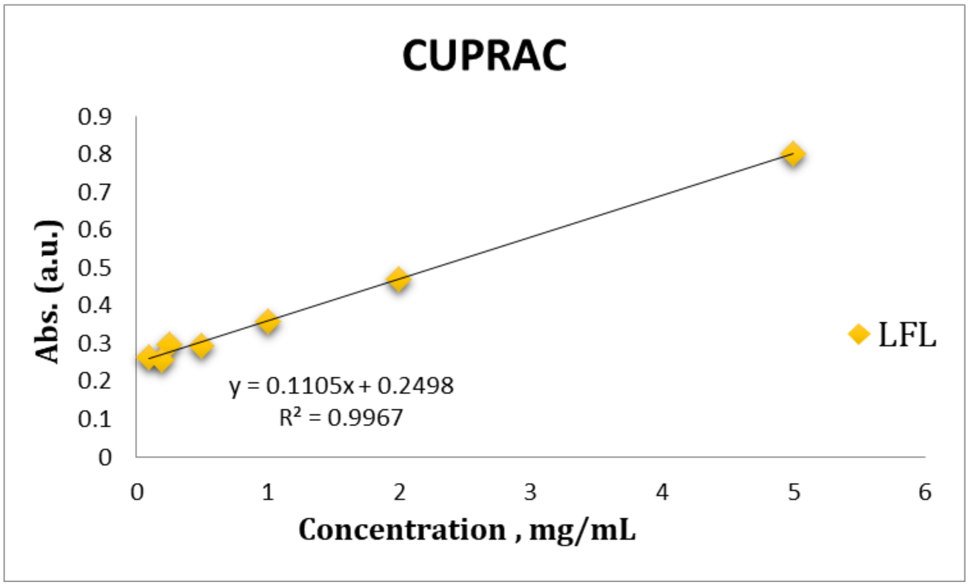

Figure S5. Dose-response curves for the AOA using DPPH, ABTS, FRAP, CUPRAC methods of the samples of honeysuckle extract (LFL) at the concentration of 0.1 – 5 mg/mL

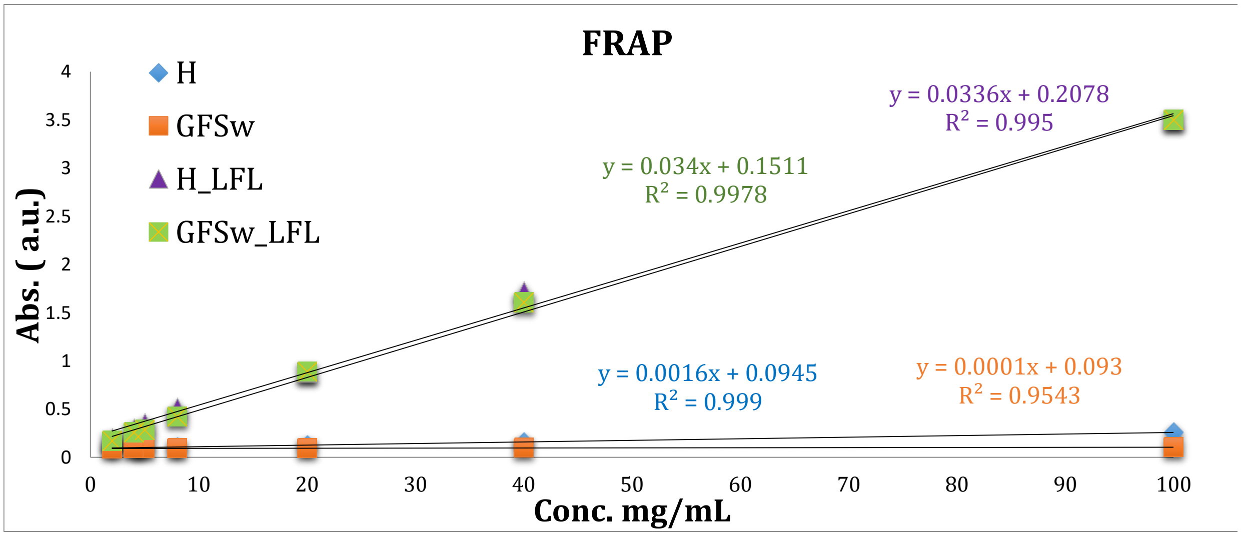
FRAP
H
GFSw
H_LFL
GFSw_LFL
y = 0.0336x + 0.2078
R² = 0.995
y = 0.034x + 0.1511
R² = 0.9978
y = 0.0016x + 0.0945
R² = 0.999
y = 0.0001x + 0.093
R² = 0.9543
Abs. (a.u.)
Conc. mg/mL

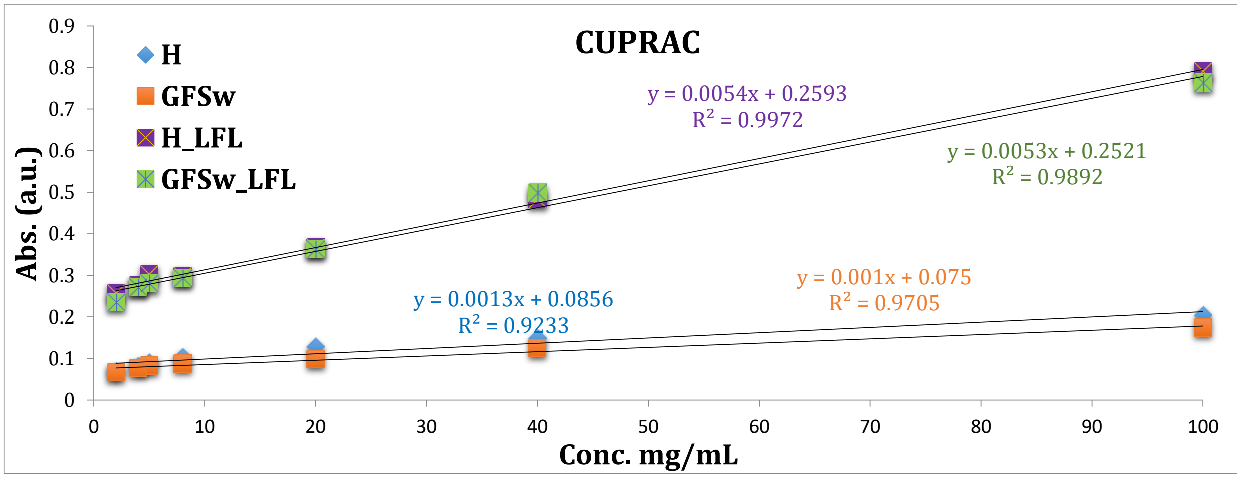
CUPRAC
H
GFSw
H_LFL
GFSw_LFL
y = 0.0054x + 0.2593
R² = 0.9972
y = 0.0053x + 0.2521
R² = 0.9892
y = 0.001x + 0.075
R² = 0.9705
y = 0.0013x + 0.0856
R² = 0.9233
Abs. (a.u.)
Conc. mg/mL

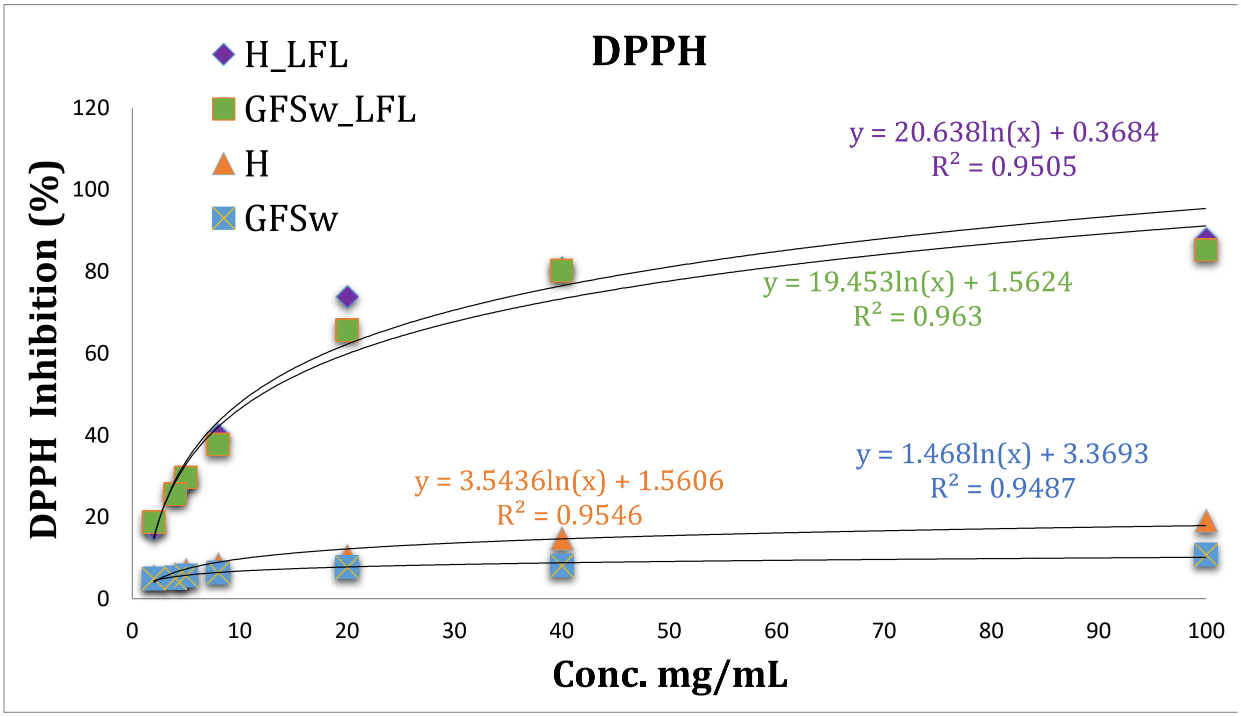

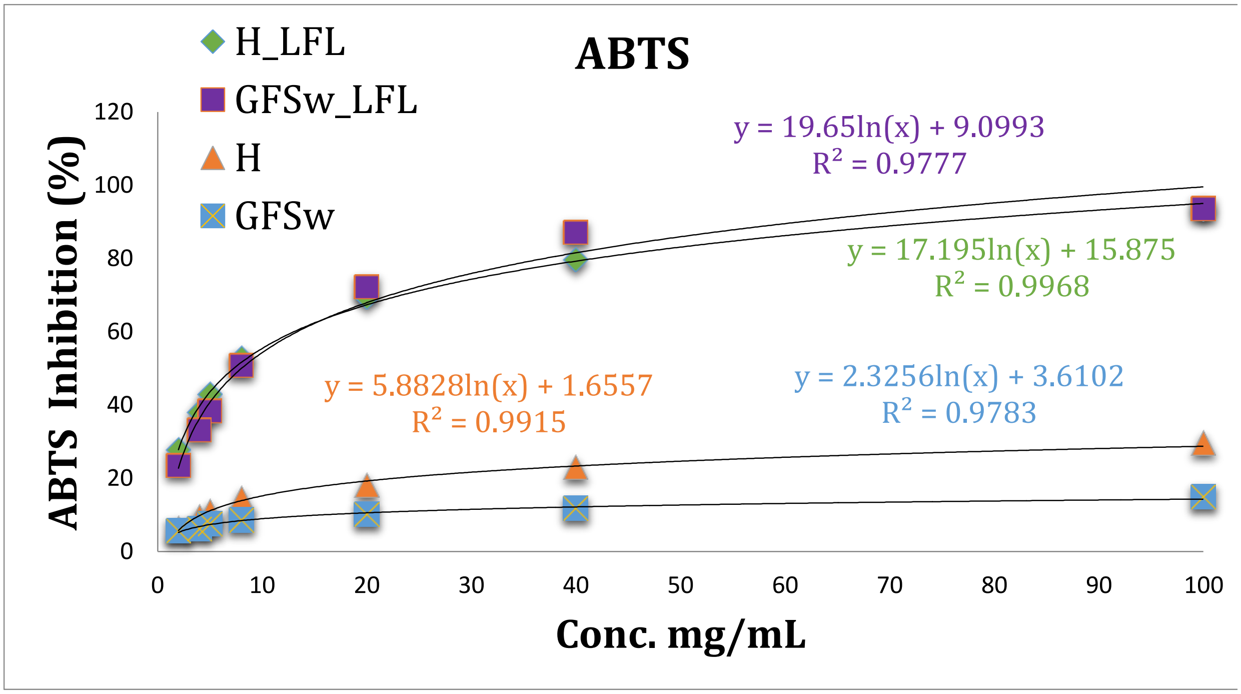

Figure S6. Dose-response curves for the AOA using DPPH, ABTS, FRAP, CUPRAC methods of the samples of honey (H), biomimetic NaDES with honey (GFSw), honey enriched with honeysuckle extract (H_LFL), GFSw enriched with honeysuckle extract (GFSw_LFL) at the concentration 2 - 100 mg/mL of the sample. These curves were used to calculate and evaluate the modulation of LFL.

Dose-Response curves of the extracts in terms of AOA by DPPH

Dose-Response curves of the honey mixtures in terms of AOA by DPPH

Dose-Response curves of the GFSw mixtures in terms of AOA by DPPH

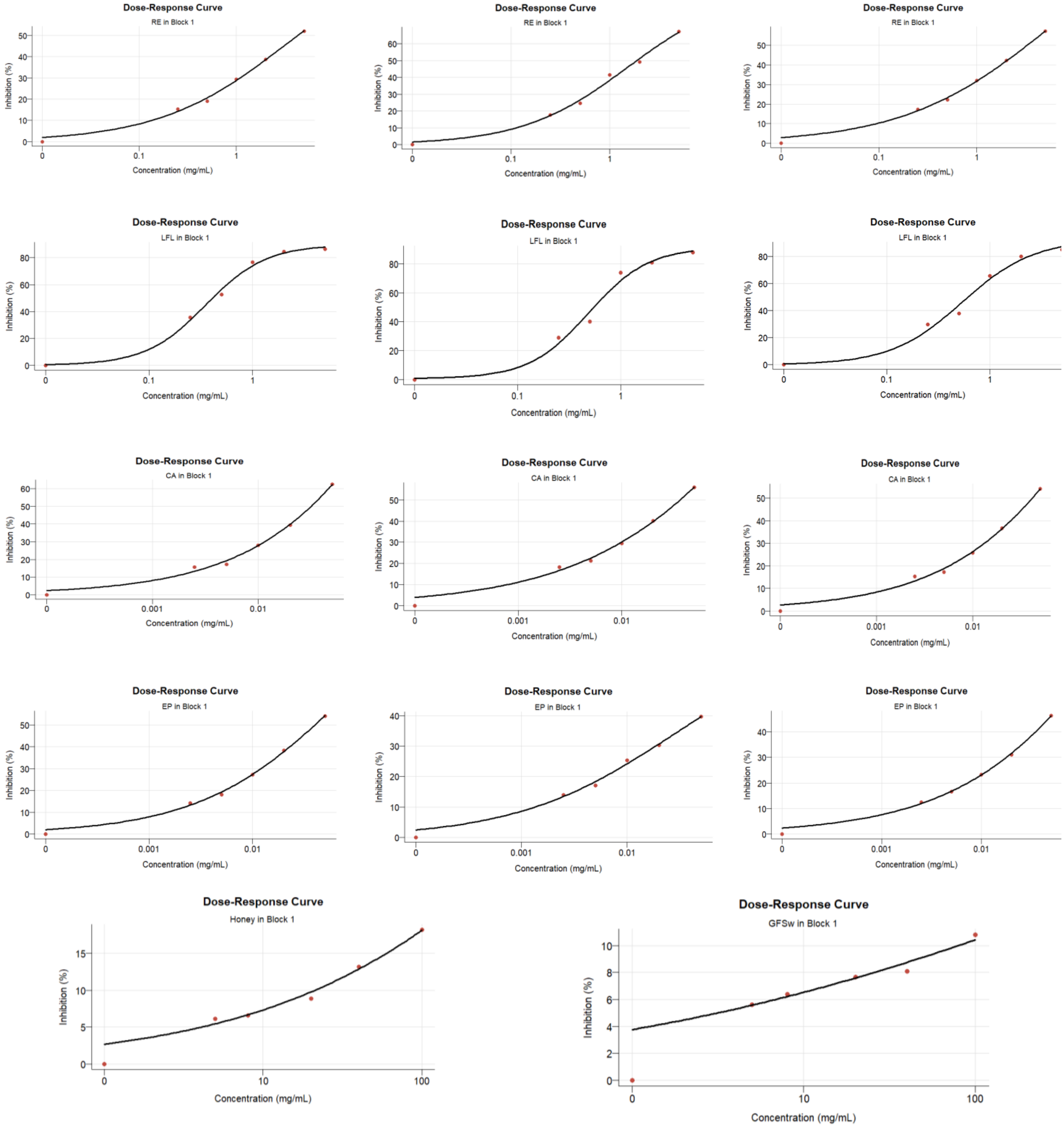

Figure. S7 . Dose-response curves of the samples generated by SynergyPlot in terms of AOA measured by DPPH: LFL – honeysuckle flower extract, RE – rasberry extract, CA – caffeic acid, EP – epicatechin, AOA –antioxidant activity as a function of polyphenols concentration

Dose-Response curves of the extracts in terms of AOA by ABTS

Dose-Response curves of the honey mixtures in terms of AOA by ABTS

Dose-Response curves of the GFSw mixtures in terms of AOA by ABTS

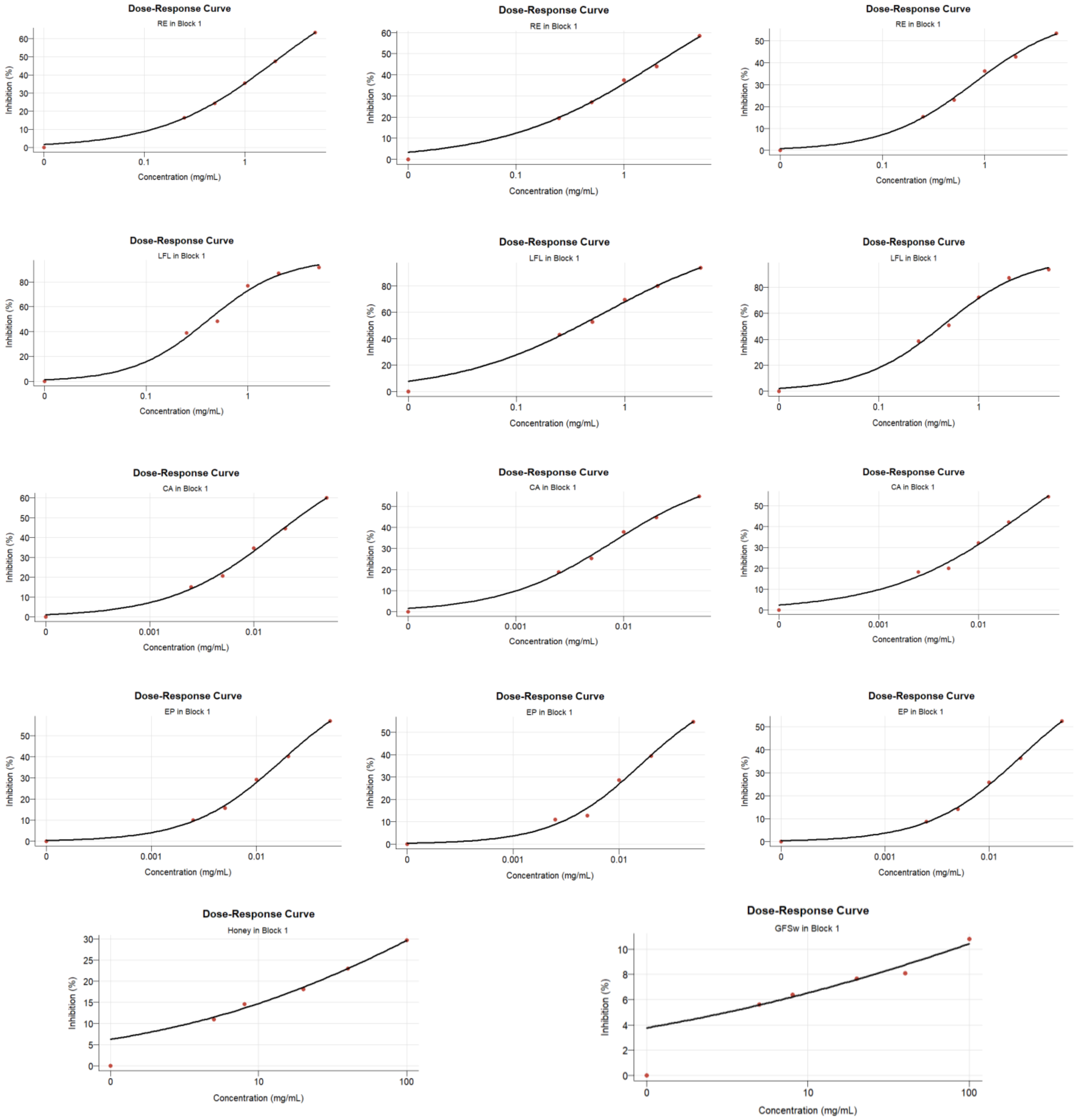

Figure. S8. Dose-response curves of the samples generated by SynergyPlot in terms of AOA measured by ABTS: LFL – honeysuckle flower extract, RE – rasberry extract, CA – caffeic acid, EP – epicatechin, AOA –antioxidant activity activity as a function of polyphenols concentration

Table S1. The correlation of AOA behaviour in terms of CI

H / GFSw	LFL	RE	CA	EP
FRAP	+1 / +1	0 / 0	+1 / 0	-1 / -1
CUPRAC	-1 / -1	0 / -1	-1 / -2	-2 / -2
DPPH IC_{50}	-1 / -1	+2 / 0(+1)	-1 / -2	-3 / -2
DPPH IC_{20}	-1 / -2	+2 / +2	+1 / 0	-1 / -1
ABTS IC_{50}	0 / 0	-3 / -3	-2 / -2	-1 / -1
ABTS IC_{20}	0(+1) / 0(+1)	+1 / 0(+1)	+1 / +1	-1 / -1

The colour code highlights: (green colour) highly similar behaviour between honeysuckle extract (LFL) and caffeic acid (CA) and epicatechin (EP) in honey (H); (turquoise colour) highly similar behaviour between LFL and RE in the biomimetic NaDES (GFSw). The blue cells indicate similar behaviour between honey and GFSw; The numbers in brackets indicate the tendency towards that value.
